# Cross-sectional personal network analysis of adult smoking in rural areas

**DOI:** 10.1098/rsos.241459

**Published:** 2024-11-27

**Authors:** Bianca-Elena Mihăilă, Marian-Gabriel Hâncean, Matjaž Perc, Jürgen Lerner, Iulian Oană, Marius Geantă, José Luis Molina, Cosmina Cioroboiu

**Affiliations:** ^1^ Department of Sociology, University of Bucharest, Panduri 90-92, Bucharest 050663, Romania; ^2^ Center for Innovation in Medicine, Theodor Pallady Boulevard 42J, Bucharest 032266, Romania; ^3^ Faculty of Natural Sciences and Mathematics, University of Maribor, Koroška cesta 160, Maribor 2000, Slovenia; ^4^ Community Healthcare Center Dr Adolf Drolc Maribor, Vošnjakova ulica 2, Maribor 2000, Slovenia; ^5^ Complexity Science Hub Vienna, Josefstädterstraße 39, Vienna 1080, Austria; ^6^ Department of Physics, Kyung Hee University, 26 Kyungheedae-ro, Dongdaemun-gu, Seoul, Republic of Korea; ^7^ Department of Computer and Information Science, University of Konstanz, Konstanz 78457, Germany; ^8^ GRAFO, Department of Social and Cultural Anthropology, Universitat Autònoma de Barcelona, Bellaterra, Barcelona 08193, Spain

**Keywords:** network science, human behaviour, data science, smoking

## Abstract

Research on smoking behaviour has primarily focused on adolescents, with less attention given to middle-aged and older adults in rural settings. This study examines the influence of personal networks and sociodemographic factors on smoking behaviour in a rural Romanian community. We analysed data from 76 participants, collected through face-to-face interviews, including smoking status (non-smokers, current and former smokers), social ties and demographic details. Multilevel regression models were used to predict smoking status. The results indicate that social networks are essential in shaping smoking habits. Current smokers were more likely to have smoking family members, reinforcing smoking within familial networks, while non-smokers were typically embedded in non-smoking environments. Gender and age patterns show that women were less likely to smoke, and older adults were more likely to have quit smoking. These findings suggest that targeted interventions should focus not only on individuals but also on their social networks. In rural areas, family-based approaches may be particularly effective due to the strong influence of familial ties. Additionally, encouraging connections with non-smokers and former smokers could help disrupt smoking clusters, supporting smoking cessation efforts.

## 1. Introduction

Tobacco smoking remains the leading cause of preventable diseases [1] and the top contributor to premature deaths in the European Union (EU) [2]. EU measures include regulations like the Tobacco Products Directive (2014/40/EU) and campaigns such as *Ex-Smokers are Unstoppable* (2014–2016). The last EU informational campaign was in 2016, with subsequent evaluations recommending SMART goals for future campaigns [3]. Since then, the focus has shifted to national campaigns, with little known about their effectiveness.

The World Health Organization reports alarming trends, with tobacco killing over 8 million people annually and affecting 1.3 million non-smokers through second-hand smoke [4]. Smokers face significantly higher risks of cardiovascular diseases, with mortality rates nearly tripling compared with non-smokers [5]. Smoking is also linked to diabetes [6], cataracts [7], gastrointestinal diseases [8] and various cancers, particularly lung cancer [9,10].

Smoking rates vary across EU regions: 28% in eastern Europe and 20% in northern Europe [11]. In 2019, Eurostat data revealed that 22.3% of men and 14.8% of women aged 15 and older smoked daily [12]. Additionally, 14% of individuals aged 15–24 were daily smokers [13]. Despite progress, current strategies are insufficient to reduce smoking incidence by 30% by 2025 [14].

Most of the tobacco literature has largely overlooked adults and older people, focusing instead on younger populations. This highlights a critical gap, underscoring the urgent need for research that includes these demographics. Such studies are vital for comprehensively understanding smoking behaviours across all age groups. This ensures that interventions and policies are well-informed and effectively tailored to meet the needs of the entire population. Additionally, rural populations have also been neglected despite evidence suggesting higher smoking rates in these areas [15]. The very few studies on rural communities emphasize smoking trends among females without focusing on gender-specific strategies [16]. We want to emphasize that most of these studies are very specific and refer to rural areas in the United States [15,17].

To address these gaps, our aim is to expand the understanding of smoking behaviours by studying often overlooked segments of the population in Romania, a representative country of Eastern Europe. Specifically, our study focuses on adults living in rural areas to bridge significant gaps identified in the existing body of literature. We investigate the relationship between social networks and smoking habits, examining how the intricate pattern of interpersonal ties within rural communities is associated with smoking behaviour. This approach not only adheres to the necessity for inclusivity in research, but also emphasizes the multifaceted interactions present in rural environments, particularly in the Eastern European context.

Romania has a high smoking prevalence: 30.6% of men and 7.5% of women smoke [18]. Among 15- to 24-year-olds, 10.2% smoke daily and 9.8% occasionally [18]. Additionally, 71.5% of daily smokers light up within 30 min of waking [19]. Literature on Romanian smoking primarily focuses on adolescents. Researchers highlight the significant impact of paternal smoking on adolescent behaviour [20], while other studies show that friends, especially best friends, significantly influence high school students’ smoking habits [21]. Furthermore, adolescents with many smoking classmates are nine times more likely to smoke [22]. Other researchers emphasize the strong correlation between adolescents’ smoking habits and those of their close peers [23].

In our study, we analyse three distinct groups—current smokers, former smokers and non-smokers—to understand smoking behaviour among the adult population in rural Eastern Europe and to inform new prevention strategies. Most existing studies have focused exclusively on current smokers [24,25], with only a few discussing the initiation of smoking by former smokers or non-smokers due to their personal network connections [26]. For example, according to Christakis & Fowler [27], smoking behaviours, both initiation and cessation, can spread through social networks. We propose that assortativity—a concept indicating that people with similar traits (e.g. smoking status) are more likely to share social ties—provides a valuable analytical framework for understanding the clustering patterns observed in smoking behaviour [28,29]. *Assortativity* does not separate *social selection* (e.g. people who smoke prefer to interact with people who smoke), *peer socialization* or *social influence* (e.g. people adopt smoking as a result of interacting with those who smoke) and *context* (*confounding*), where external factors related to the environment or setting influence network formation.

Studies identify influential family ties (parents and siblings) and friends. Some find peer influence outweighs parental influence [30–32], while others argue parental influence is equal or greater [33,34]. These studies underscore the importance of social networks and context in shaping smoking behaviour. Understanding the roles of peers and family is crucial for controlling smoking behaviours. Personal networks (individuals and their direct social contacts) constitute the immediate social context that influences people. Examining the structure (how people are connected) and composition (with whom people interact) of social networks is useful for modelling smoking behaviours. The literature highlights that a dense social network can reinforce group behavioural norms [35]. Opinion leaders increase their influence as their centrality in networks grows [35,36]. Isolated adolescents are more likely to smoke [37,38], suggesting that fewer social connections correlate with higher smoking rates. Conversely, individuals in denser social networks are more likely to share similar smoking behaviours due to shared attitudes or social control within the network.

In our study, we explored the clustering of tobacco use within personal networks in a rural Eastern European context, deploying a personal network research design [39]. A *personal network* includes a focal individual (*ego*), their direct social contacts (*alters*), and the specific patterns of connections among those alters. This methodology enables us to study how social tie arrangements affect smoking habits in rural areas, filling crucial research gaps and highlighting local dynamics. We evaluated quantitative cross-sectional data from adults over 18 in Lerești, a small rural village in Argeș County, Romania. We analysed data on 76 people’s personal networks (egos), social contacts (alters) and alter connections to study network impacts on middle-aged and older adults. Our hypothesis was that smoking behaviours (e.g. active smoking) are more likely when peers (social contacts) exhibit similar smoking behaviours. The research objective was to assess the extent to which assortativity predicts patterns of tobacco use in these rural settings.

## 2. Material and methods

### 2.1. Research design and terminology

Social network analysis (SNA) is a mixed-methods framework for investigating social relations, supported by a robust theoretical foundation [40] and comprehensive research tools [41]. There are two main approaches to studying relational patterns. The first explores and tests explanations for the generative processes of networks, identifying factors that explain observed patterns [42]. The second assesses the impact of relational patterns on outcomes such as health [43,44], idea spread [45], capitalization [46] and employment opportunities [47].

Methodologically, there are three research designs for collecting network data. First, the socio-centric approach depicts relationships among individuals in the same social unit, such as pupils in a classroom [48] or colleagues in a workplace [49]. Second, the ego-centric approach explores an individual’s relationships within a socio-centric network. Third, the personal network approach samples data from multiple social environments in which a respondent is embedded [39].

In SNA, relationships are referred to as social ties, with networks consisting of nodes (individuals) and their ties. In socio-centric or ego-centric designs, data are collected from each node in the network, similar to connecting points in a graph. In personal network analysis (PNA), respondents (*egos*) provide information about themselves, their social contacts (*alters*) and the relationships among these contacts (*alter–alter* ties). Alters are sampled from various social circles in which the ego is embedded. Unlike other approaches, PNA typically produces networks based solely on information from the egos [50,51].

### 2.2. Setting

We interviewed 83 people selected from a small rural community in Romania. Due to incomplete data, we removed seven study participants and kept the rest of the 76 individuals in the analysis. We employed a PNA research design [39]. Data were collected from study participants (dubbed *egos*). Egos reported information about themselves and nominated social contacts (*alters*) based on a name-generator question. They provided information about each alter’s characteristics, *alter–alter* ties and *ego–alter* ties. There are two types of information: *attribute data* (characteristics of egos and alters) and *network* (structural) *data* (how social contacts and egos are embedded in the personal network).

The size of network generators (i.e. how many people a respondent is asked to nominate) has been a research topic. Research suggests the number of elicited alters should be between 30 and 60 for better network property capture [51–53]. Working with name generators of at least 40 imposes practical problems in terms of data collection. As the number of alters increases, the respondent burden [50,54] also increases, with all its subsequent effects. Recent work shows that 25 alters is a good trade-off between a good estimation of structural properties and respondent burden [55].

The data collection was part of a fieldwork research conducted in Lerești (a small rural community), Argeș County, Romania. We gathered the data between 13 and 30 September 2023. The study data are unique, and come from a cohort of respondents that were included in a Living Lab. We plan to observe this group of study participants at multiple points in time during 2023–2025. The concept of a Living Lab does not have a widely recognized definition [56]. Nevertheless, the European Network of Living Labs (ENoLL), an umbrella organization for all the Living Labs around the world, defines it as: ‘user-centered open innovation ecosystems based on a systematic user co-creation approach, integrating research and innovation processes in real-life communities and setting’ [57].

We deployed PNA because this network research design best fits our objective of assessing whether the smoking behaviour of the ego correlates with the smoking behaviour of their elicited social contacts. Given the rural setting, PNA allows for the collection of network data while respecting participants’ privacy, as it focuses on egos’ perceptions and reported interactions rather than mapping the entire community network.

### 2.3. Data collection

**Figure 1.**
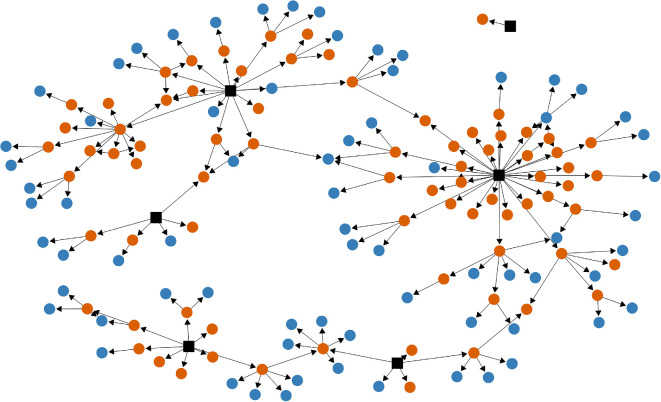
Link-tracing sampling. The black squares represent the initial individuals, referred to as ‘seeds’, who were invited by researchers to participate in the study. The circles depict individuals who were recommended by the study’s participants. Those marked in red indicate individuals who participated in the study, while the ones in blue signify individuals who did not participate for various reasons, such as refusal or the need to reschedule. The directional arrows within the figure demonstrate the flow of the referral process, tracing the path from referees to their referrals.

We deployed a link-tracing sampling design (see figure 1) to recruit the study participants [58–60]. We identified the participants in a chain-referral fashion: interviewed participants recommend other interested individuals. Based on ethnographic fieldwork notes, our recruitment commenced with six diverse seeds (initial participants) to ensure a heterogeneous mix covering a broad spectrum of socio-economic indicators and age groups, from young to older individuals. It is important to note that our sample predominantly consisted of middle-aged and older adults. This demographic skew is reflective of the population age structure in Lerești, Argeș County, Romania, where the data collection took place, rather than a methodological bias. Following the data provided by National Institute of Statistics, out of the 4124 residents in Lerești, 1965 are aged 50 or older (48%) [61]. Thus, our seeds were different in sex (four males and two females), age (*range* = 30, *min.* = 34, *max.* = 64), employment sector (one retired, three employed in public sector, one employed in the private sector and one self-employed) and education (five with BA studies and one with high-school diploma). Two seeds declined to participate as interviewees but provided recommendations for participation. Face-to-face interviews were collected between 13 and 23 September 2023, in Lerești, Romania.

We conducted the interviews using the Network Canvas software package [62], an advanced tool designed specifically for surveying personal networks. Respondents actively participated in the interview process, with data entry occurring in real time within the software. This approach ensured that participants were engaged throughout the data input process, allowing them the opportunity to monitor and verify the information being recorded at any moment. The average interview lasted approximately 80 minutes. Instead of financial incentives, participants were given access to a hotline for medical second opinions and inquiries about disease prevention and healthy lifestyles. Our study followed the recommendations, guidelines and regulations of the Romanian Sociologists Society (i.e. the professional association of Romanian sociologists) and the Declaration of Helsinki. The research protocol was approved by a named institutional/licensing committee. Specifically, the Ethics Committee of the Center for Innovation in Medicine (InoMed) reviewed and approved all these study procedures (EC-INOMED Decision No. D001/09-06-2023 and No. D001/19-01-2024). All participants gave written informed consent. The privacy rights of the study participants were observed. The authors did not have access to information that could identify participants. After each interview, information that could identify the participants was anonymized. Before conducting the interview, we provided each participant with a dossier containing informative materials about the project’s objectives, how the data would be analysed and reported, and their participation rights (e.g. the right to withdraw from the project at any time, even after the interview was completed).

### 2.4. The questionnaire

The questionnaire was structured in several blocks. (1) *Sociodemographic variables survey*: We surveyed egos on various sociodemographic variables, including sex assigned at birth, age, marital status, education level, employment status and smoking status (smokers, occasional smokers, former smokers, non-smokers and never smokers). (2) *Alter generator*: Next, we incorporated an alter generator to identify individuals from the ego’s close network. We requested each ego to nominate nearly 25 alters (aged 18 years or older) with whom they frequently interact and feel emotionally close. This aimed to include both close circle individuals and broader personal network ties, ensuring sufficient data for statistical modelling [63]. (3) *Name interpreter*: Egos provided sociodemographic details and smoking statuses of the nominated alters, using similar response options as for egos. (4) *Connections between alters*: Egos assessed whether their alters know and communicate with each other in the ego’s absence. (5) *Intensity of ego–alter ties*: We assessed the intensity of ego–alter ties by asking about the frequency of their communications (ranging from more than once a week to less than once a year) and their level of emotional closeness (from ‘not close at all’ to ‘very close’). We also created a binary variable for meeting frequency (at least weekly and less than weekly). Prompts included: ‘How often do you typically meet with each of the people you previously mentioned?’ and ‘How emotionally close do you feel to the people you previously mentioned?’.

### 2.5. Attribute data

The study participants provided information regarding their sex (assigned at birth: male or female), date of birth and the highest level of educational attainment. This latter was coded as a binary variable, with 0 indicating ‘no university degree’ and 1 indicating ‘possession of a university degree’. Employment status was identified as *employed*, *unemployed* or *retired* and subsequently coded as a binary variable, with 0 representing ‘unemployed’ (this category includes retired individuals) and 1 representing ‘employed’. Marital status was recorded as *single*, *married* or *in a relationship*, and later coded as a binary variable: 0 for ‘single’ and 1 for ‘in a relationship’ (this includes married individuals).

Participants were also asked about their smoking habits, with options to identify as *smokers*, *occasional smokers*, *former smokers*, *non-smokers* (those who have smoked too infrequently to be considered regular smokers) and *never smokers*. For analysis, these responses were later grouped into three categories: *smokers* (encompassing both regular and occasional smokers), *former smokers* and *non-smokers* (individuals who have smoked minimally or have never smoked). Participants also self-reported any medical conditions (yes/no) and their participation in sports activities (yes/no). Additionally, they were asked about the occurrence of cancer or cardiovascular diseases among immediate family members (yes/no). Participants provided details about their social contacts (alters), including each alter’s age, sex (assigned at birth: male or female), the highest level of education, and whether the alter adheres to a specific diet or engages in sports as a hobby (yes/no), and their smoking status (measured the same as for egos).

### 2.6. Structural and compositional data

For each personal network, we assessed composition by calculating the *proportion of alters* categorized as smokers, former smokers and non-smokers, which indicates the predominance of smoking behaviours within the network. We then calculated *network density*, the ratio of actual to possible connections, to gauge the potential speed of behaviour and information transmission. Lower-density networks may lack interconnectedness, possibly leading to insufficient social support for healthy behaviours.

Additionally, we identified *network components*, which are sub-networks where members are interconnected but disconnected from other sub-networks. Recognizing components with higher incidences of specific smoking behaviours allows for tailored intervention strategies. Lastly, we measured *network centralization* to understand how smoking behaviours propagate through the network, focusing on key central nodes for targeted influence.

In our study, the *assortativity variable* is the variable of interest. This variable enabled us to examine the likelihood of alters’ smoking behaviours being influenced by the smoking habits of their connected peers. We calculated this variable for each alter within our personal networks, employing the formula established in prior studies on assortativity related to COVID-19 vaccine opinions [64] and dietary patterns [65]. Specifically, assortativity was determined by calculating the difference between the proportion of an alter’s neighbours who smoke and the overall proportion of smokers within the ego network, excluding the alter in question from this calculation. We applied the same method to compute assortativity for former smokers and non-smokers. Each category of smoking status was dichotomized (where 1 represents smokers/former smokers/non-smokers and 0 encompasses all other statuses). These computations allow us to test the hypothesis that an alter’s smoking behaviour is associated with the smoking patterns of their immediate social circle. For our analysis of the 76 ego networks, we excluded peers who did not have neighbours, as the assortativity measure does not apply to them.

We illustrate the calculation of the assortativity variable using smoking behaviour as an example. Consider ego *j*’s network consisting of 25 alters, including nine smokers. If we focus on a specific smoker alter *k* with four smoking neighbours, we exclude alter *k* to calculate the proportion of smokers in ego *j*’s network, which is 8 out of 24, or 0.33. Among alter *k*’s neighbours, the proportion of smokers is 4 out of 4, or 1.00. The assortativity variable for alter *k* is then 0.67, representing the difference between the smoking prevalence among alter *k*’s neighbours (1.00) and the adjusted prevalence in ego *j*’s network (0.33).

### 2.7. The statistical analysis

We used a multilevel analytic approach to predict the smoking status of alters, considering the hierarchical structure where alters (level one) are nested within egos (level two). The dependencies within networks were also accounted for in our analysis. The dependent variables were the binary-coded smoking status of alters: 1 for smokers and 0 for others; 1 for former smokers and 0 for others; 1 for non-smokers and 0 for others.

Actor-level variables for each ego included *sex* (coded 0 for male, 1 for female), *age* (years, ≥18), *education level* (separating between persons with and without university education) and *employment status*, categorizing respondents as employed and unemployed (including retired persons). *Marital status* was included in the analyses in a binary form, distinguishing between those who are single and those who are in a relationship (married or unmarried). *Smoking status* was split into three categories, separating between *smokers* (current and occasional), *non-smokers* (persons who never smoked or minimal smokers) and *former smokers*.

Since the alters were not directly interviewed, we relied on the egos’ perceptions as proxies to gather information about the alters. This approach involved asking the egos to provide details on the alters’ *sex*, *age*, *education level*, *marital status* and *smoking status*, using the following prompt: ‘Of the persons you mentioned, please tell me which of them are […]’.

Furthermore, we centralized and scaled the *age* variable for both egos and alters to improve the numerical stability of our models and to mitigate the impact of outliers. Subsequently, we calculated key structural network metrics: *density*, which reflects the proportion of actual connections relative to the maximum possible connections within the network; *degree centrality*, indicating the number of direct connections a node has with others; and *the number of components*, identifying distinct interconnected groups within the network that are isolated from each other. We also performed centralization and scaling adjustments on *degree* and *betweenness centrality* metrics to account for their varying importance across networks of different sizes.

In our analysis, we examined the personal networks of 76 egos, which included a total of 1681 alters. For each ego, we assigned a binary variable to denote their *smoking status*: 1 was used to indicate a *current smoker*, while 0 represented non-smokers and other categories. This coding scheme was applied similarly to distinguish *former smokers* and *non-smokers*. An analogous binary variable was also assigned for each alter to reflect their smoking status. Our primary objective was to deploy models to predict the smoking behaviours of alters based on these binary classifications.

We want to provide further details on the management of network size in the context of data analysis. First, we refer to the size of the alters’ networks. Alters within the personal networks vary in their number of direct connections. To control for this variation, we included the ‘alter degree’ as a variable in the multilevel logistic regression models (see §3). This effect accounts for the number of direct neighbours each alter has, ensuring that the influence of alter network size on smoking behaviour is properly controlled. The estimates of this effect illustrate whether the variation in alter network size significantly impacts the findings. Second, we refer to the size of the egos’ personal networks. From the initial sample of 83 personal networks, we retained only those networks with at least 20 alters for whom smoking status information was fully available. This threshold was chosen to ensure sufficient and consistent data for robust statistical estimation. By setting this criterion, we aimed to enhance the reliability of our analysis while minimizing potential biases from incomplete or insufficient data. Additionally, the definition of the assortativity variable, by design, normalizes with respect to ego-network size and with respect to alter degree (by considering the ratio of smokers).

In addition to the multilevel logistic regression models, we executed standard logistic general linear models (GLMs) (electronic supplementary material, tables S36, S44 and S52). The GLM results give additional confidence in the robustness of findings. From a theoretical perspective, the multilevel models (the ones we reported in the article) should be more appropriate since they do account for the nesting of observations (alters) within egos—which is a fact of the data collection process. GLMs, on the other hand, are simpler and readers are certainly more familiar with them (they are available in the electronic supplementary material). Accord between GLMs and multilevel models would point to robustness. The GLMs control for network size in the same way as the multilevel models (the definition of the assortativity variable normalizes by size: number of alters and alter degree) and additionally alter degree is used as a control variable in the models—as in the multilevel models.

## 3. Results

### 3.1. Descriptive statistics

Table 1 outlines the sociodemographic attributes of the egos. Among our 76 participants, the majority are non-smokers (either never smoked or smoked minimally) (ƒ = 32; 42.1%). The distribution by sex is balanced (ƒ = 38; 50% for both males and females), with a slight majority being part of the active workforce (ƒ = 40; 52.6%). The vast majority is in a relationship or married (ƒ = 66; 86.8%), with a median age of 55 years (range = 62.0). Educationally, most do not have a university degree (non-tertiary level) (ƒ = 44; 57.9%). Summary statistics for personal networks’ structural features show that personal networks have as a median two distinct components (median = 2.0, range = 16.0), likely reflecting family and close friends, being characterized by a low centralization score (mean = 0.4; s.d. = 0.1) and relatively low network density (mean = 0.3; s.d. = 0.2). Table 1 also provides the sociodemographic characteristics of the 1681 alters, who are predominantly female (ƒ = 886; 52.7%), either married or in a relationship (ƒ = 1347; 80.1%) and do not have a university degree (ƒ = 1001; 59.5%). Their median age is comparable to that of the egos (median = 53 years, range = 77). The table includes the frequency of meetings between ego and alters, indicating that these interactions occur at least weekly (ƒ = 948; 56.4%). The network’s structural characteristics reveal that alters generally have a low degree of connectivity within the network (mean = 0.05; s.d. = 0.03). Additionally, alters tend to play a fairly average role in linking others within the network, as indicated by the betweenness centrality score (mean = 0.04; s.d. = 0.09). Regarding the assortativity variable, the non-smoker category shows the highest standard deviation (s.d. = 0.22) compared with the former smoker (s.d. = 0.14) and smoker (s.d. = 0.20) categories. In this sense, the assortativity exhibits the most variability in the case of non-smokers.

**Table 1.** Descriptive statistics for egos and alters. This table summarizes the key demographic and network characteristics for both egos (*n* = 76) and alters (*n* = 1681). The statistics, presented as means, standard deviations and medians, are essential for understanding the variables used in the subsequent analysis of how personal networks influence smoking behaviours, which is explored further in the regression models. We performed the structural measurements on the personal networks, including the isolates.

characteristic		values for ego and network data (*n* = 76)	values for alter data (*n* = 1681)
**age**			
	age in years, mean (s.d.)	54.04 (16.1)	52 (16.1)
	age in years, median age (range)	55 (18–80)	53 (18–95)
**smoking status, *n* (%)**			
	smokers	20 (26.3%)	455 (27.1%)
	former smokers	24 (31.6%)	190 (11.3%)
	non-smokers	32 (42.1%)	1036 (61.6%)
**sex, *n* (%)**			
	male	38 (50.0%)	795 (47.3%)
	female	38 (50.0%)	886 (52.7%)
**education level, *n* (%)**			
	lower education level (no university degree)	44 (57.9%)	1001 (59.5%)
	higher education level (at least university degree)	32 (42.1%)	622 (37.0%)
	missing	0 (0.0%)	58 (3.5%)
**employment status, *n* (%)**			
	employed	40 (52.6%)	—
	unemployed (including retired people)	36 (47.4%)	—
**marital status, *n* (%)**			
	single	10 (13.2%)	334 (19.9%)
	in a relationship (married or not)	66 (86.8%)	1347 (80.1%)
**ego–alter meeting frequency, *n* (%)**			
	at least weekly		948 (56.4%)
	less than weekly		733 (43.6%)
mean components (s.d.)		3.5 (3.6)	
median components (range)		2 (1–17)	
mean degree centralization (s.d.)		0.4 (0.1)	
mean density (s.d.)		0.3 (0.2)	
mean alter degree (s.d.) (standardized)		—	0.05 (0.03)
mean alter betweenness (s.d.) (standardized)		—	0.04 (0.09)
alters’ assortativity score for former smoker status		—	0.01 (0.14)
alters’ assortativity score for non-smoker status		—	−0.01 (0.22)
alters’ assortativity score for smoker status		—	0.02 (0.20)

Table 2 illustrates the distribution of egos by their smoking status and various demographic variables. Namely, the distribution of smokers is balanced across sex (ƒ = 10; 50.0%). A significant proportion of smokers (ƒ = 18; 90.0%) report being in a relationship, with an equal division across educational attainment levels, categorized as lower (no university degree) and higher education (at least a bachelor’s degree). Furthermore, the majority of these individuals are employed (ƒ = 15; 75.0%). The mean age for smokers among the egos is the lowest across all groups, averaging 49.1 years. The table also includes the type of relationship that the ego has with each nominated alter (i.e. family member, close friend, simple friend or acquaintance). The results indicate that, regardless of the ego’s smoking status, most connections are with alters who are considered family members.

**Table 2.** Descriptive statistics based on smoking status of egos. This table presents the demographic characteristics of egos, grouped by their smoking status: current and occasional smokers, former smokers and non-smokers. The data include age, sex, education level, employment status and marital status, as well as the type of relationship egos have with their alters. These statistics provide insights into the sociodemographic patterns associated with each smoking category. For instance, former smokers are predominantly male, older and more likely to be unemployed compared with current smokers. The high proportion of family members among non-smokers highlights the potential influence of familial relationships on smoking cessation or prevention.

characteristic		egos’ smoking status		
egos		smoker	former smoker	non-smoker
**age in years, mean (s.d.)**		49.1 (13.8)	54.7 (16.2)	56.6 (17.2)
**age in years, median age (range)**		51.5 (18-68)	58 (22–80)	65.5 (21-76)
**sex, *n* (%)**				
	male	10 (50.0%)	19 (79.2%)	9 (28.1 %)
	female	10 (50.0%)	5 (20.8%)	23 (71.9%)
**education level, *n* (%)**				
	lower education level (no university degree)	10 (50.0%)	14 (58.3%)	20 (62.5%)
	higher education level (university degree)	10 (50.0%)	10 (41.7%)	12 (37.5%)
**employment status**				
	unemployed (including retired people)	5 (25.0%)	13 (54.2%)	18 (56.2%)
	employed	15 (75.0%)	11 (45.8%)	14 (43.8%)
**marital status, *n* (%)**				
	single	2 (10.0%)	3 (12.5%)	5 (15.6%)
	in a relationship (including married)	18 (90.0%)	21 (87.5%)	27 (84.4%)
**type of ego relationship with alters**				
	family member	216 (48.5%)	226 (41.8%)	394 (56.7%)
	close friend	118 (26.5%)	107 (19.8%)	115 (16.5%)
	simple friend	93 (20.9%)	128 (23.7%)	128 (18.4%)
	acquaintance	18 (4.0%)	80 (14.8%)	58 (8.3%)

Among the egos who are former smokers, a significant majority are male (ƒ = 19; 79.2%) and are currently in a relationship (ƒ = 21; 87.5%). A notable proportion is unemployed (or retired) (ƒ = 13; 54.2%) and possesses a lower educational attainment (ƒ = 14; 58.3%). When compared to current smoking egos, former smokers exhibit a higher median age (median = 58 years, range = 58.0). Similarly, within the alter group (table 3), former smokers are predominantly males (ƒ = 134; 70.5%). Most possess lower educational qualifications (ƒ = 122; 64.2%) and are in a relationship (ƒ = 164; 86.3%). The average age among alters who have quit smoking stands at 55.6 years. This aligns with research indicating an increased likelihood of smoking cessation with advancing age [66,67].

**Table 3.** Descriptive statistics based on smoking status of alters. This table presents the demographic characteristics of alters, grouped by smoking status: current and occasional smokers, former smokers and non-smokers. The variables include age, sex, education level, marital status and frequency of meetings with egos. The data show differences in smoking categories, with a higher proportion of males among smokers and former smokers. Former smokers are generally older, more likely to be in relationships and tend to meet with egos more frequently.

characteristic		alters’ smoking status		
alters		smoker	former smoker	non-smoker
**age in years, mean (s.d.)**		46.9 (14.4)	55.6 (13.3)	53.5 (16.8)
**age in years, median age (range)**		50 (18–82)	56 (21–85)	55 (18–95)
**sex, *n* (%)**				
	male	253 (55.6%)	134 (70.5%)	408 (39.4%)
	female	202 (44.4%)	56 (29.5%)	628 (60.6%)
**education level, *n* (%)**				
	lower education level (no university degree)	265 (58.2%)	122 (64.2%)	614 (59.3%)
	higher education level (university degree)	172 (37.8%)	64 (33.7%)	386 (37.3%)
	missing	18 (4.0%)	4 (2.1%)	36 (3.5%)
**marital status, *n* (%)**				
	single	93 (20.4%)	26 (13.7%)	215 (20.8%)
	in a relationship (including married)	362 (79.6%)	164 (86.3%)	821 (79.2%)
**ego–alter meeting frequency, *n* (%)**				
	less than weekly	200 (44.0%)	73 (38.4%)	460 (44.4%)
	at least weekly	255 (56.0%)	117 (61.6%)	576 (55.6%)

According to table 2, the majority of our non-smoking participants are female (ƒ = 23; 71.9%) and either married or in a relationship (ƒ = 27; 84.4%). When it comes to educational attainment, a significant portion primarily falls within the lower education category (ƒ = 20; 62.5%). Among the never-smoking egos, this group emerges as the oldest, with an average age of 56.6 years and a median age of 65.5 years, spanning an age range of 21–76 years. This trend may suggest that the propensity to never smoke or to smoke minimally escalates with advancing age. Similarly, never-smoking alters (table 3) display a mean age of 53.5 years and a median age of 55 years, with their ages varying from 18 to 95 years. This indicates that, akin to their ego counterparts, alters who have never smoked also tend to be older, albeit with a wide age distribution.

In alignment with ego data, the data from table 3 show that a substantial majority of the smoking alters are also in a relationship (ƒ = 362; 79.6%), with a significant number having achieved a lower education level (non-university education) (ƒ = 265; 58.2%), whereas 37.8% (*f* = 172) have attained higher education (at least a bachelor’s degree). Smoking alters exhibit a slightly younger demographic compared to smoking egos, with a median age of 50 years (range = 64.0) versus the smoking egos’ median age of 51.5 years (range = 50.0). When examining the frequency of interactions between alters and egos, it becomes evident that the majority of alters, irrespective of their smoking status, tend to meet with egos at least on a weekly basis.

### 3.2. Model estimation

We conducted a multilevel regression analysis, creating separate models for each smoking category. The structure of the analysis comprises four distinct models: Model 0, which establishes a baseline for comparisons; Model 1, incorporating individual attributes; Model 2, examining network characteristics; and Model 3, a comprehensive model that integrates both individual attributes and network characteristics. Table 4 reports the results of fitting models that predict alters’ smoking behaviour. The dependent variable in our analysis is the smoking status of the alter, coded as a binary variable (smoker versus all other categories). For smokers, the findings indicate a reduced likelihood of smoking associated with being female (Model 1, odds ratio (OR) = 0.61; 95% confidence interval (CI): 0.48, 0.78, *p* < 0.001; Model 3, OR = 0.60; CI: 0.47, 0.77, *p* < 0.001) and increased age (Model 1, OR = 0.68; 95% CI: 0.59, 0.78, *p* < 0.001; Model 3, OR = 0.68; CI: 0.59, 0.79, *p* < 0.001).

**Table 4.** Multilevel logistic regression models predicting alters’ smoking status. This table shows the results of multilevel logistic regression models predicting whether alters are smokers. The models evaluate the influence of individual attributes (Model 1), network characteristics (Model 2) and a combination of both (Model 3). The results highlight the significant role of social networks, particularly smoking family members, in influencing alters’ smoking behaviour. The assortativity of smokers within the network and the presence of smoking close friends are strong predictors of smoking status.

	Model 0 (‘intercept’)		Model 1 (‘attributes’)		Model 2 (‘network’)		Model 3 (‘full’)	
	**OR (CI**)	***p***	**OR (CI**)	***p***	**OR (CI**)	***p***	**OR (CI**)	***p***
alter sex [ref. = male]			**0.61**	**<0.001**			**0.60**	**<0.001**
			**0.48, 0.78**				**0.47, 0.77**	
alter age (mean centred, scaled)			**0.68**	**<0.001**			**0.68**	**<0.001**
			**0.59, 0.78**				**0.59, 0.79**	
alter education [ref. = lower education]			0.97	0.793			1.00	0.998
			0.74, 1.26				0.77, 1.31	
alter marital status [ref. = single]			0.95	0.742			0.96	0.780
			0.70, 1.29				0.70, 1.31	
ego sex [ref. = male]			0.86	0.386			0.81	0.235
			0.61, 1.21				0.57, 1.15	
ego age (mean centred, scaled)			0.95	0.585			0.92	0.403
			0.79, 1.14				0.75, 1.12	
ego education [ref. = lower education]			0.73	0.075			0.72	0.087
			0.51, 1.03				0.50, 1.05	
ego marital status [ref. = single]			0.93	0.763			0.95	0.844
			0.57, 1.51				0.56, 1.60	
ego employment [ref. = unemployed]			1.17	0.426			1.15	0.500
			0.80, 1.70				0.77, 1.70	
acquaintance smoker [ref. = other]			2.61	0.095			2.55	0.109
			0.85, 8.02				0.81, 7.98	
simple friend smoker [ref. = other]			1.52	0.154			1.45	0.226
			0.85, 2.70				0.79, 2.65	
close friend smoker [ref. = other]			**2.61**	**<0.001**			**2.47**	**0.001**
			**1.55, 4.40**				**1.44, 4.23**	
family member smoker [ref. = other]			**2.64**	**<0.001**			**2.51**	**<0.001**
			**1.73, 4.04**				**1.62, 3.91**	
ego alter meeting frequency [ref. = less than weekly]					0.99	0.946	1.07	0.606
					0.77, 1.28		0.82, 1.41	
alter degree (mean centred, scaled)					1.07	0.359	1.01	0.882
					0.92, 1.25		0.86, 1.19	
alter betweenness (mean centred, scaled)					1.00	0.951	1.02	0.811
					0.87, 1.16		0.88, 1.18	
network components					1.00	0.986	1.04	0.349
					0.92,1.09		0.96, 1.12	
network degree centralization					0.91	0.908	1.00	0.999
					0.19, 4.33		0.24, 4.11	
network density					3.28	0.101	1.68	0.426
					0.79, 13.60		0.47, 6.03	
assortativity (smokers)					**3.44**	**<0.001**	**3.01**	**0.001**
					**1.85, 6.37**		**1.61, 5.62**	
assortativity (former smokers)					1.83	0.198	1.57	0.334
					0.73, 4.56		0.63, 3.91	
intercept	0.34	**<0.001**	0.42	**0.003**	0.22	**0.010**	0.30	**0.030**
	0.28, 0.41		0.24, 0.75		0.07, 0.70		0.10, 0.89	
num.obs	1622		1622		1622		1622	
num.groups: ego_id	76		76		76		76	
ICC	0.13		0.06		0.12		0.07	
AIC	1836.164		1775.478		1831.758		1777.152	
BIC	1846.947		1856.349		1885.672		1901.155	
log likelihood	−916.082		−872.739		−905.879		−865.576	
deviance	1832.164		1745.478		1811.758		1731.152	
marginal *R*^2^/conditional *R*^2^	0.000/0.127		0.117/0.173		0.026/0.143		0.131/0.188	

The presence of a family member who smokes (Model 1, OR = 2.64; 95% CI: 1.73, 4.04, *p* < 0.001; Model 3, OR = 2.51; CI: 1.62, 3.91, *p* < 0.001) or a close friend who smokes (Model 1, OR = 2.61; 95% CI: 1.55, 4.40, *p* < 0.001; Model 3, OR = 2.47; CI: 1.44, 4.23, *p* < 0.05) significantly increases the likelihood of an individual being a smoker. Among network-related variables, the *assortativity score* (*smokers*) stands out as the sole significant predictor, demonstrating a consistent positive relationship across the models (Model 2, OR = 3.44; 95% CI: 1.85, 6.37, *p* < 0.001; Model 3, OR = 3.01; CI: 1.61, 5.62, *p* < 0.001). This finding suggests that alters sharing similar smoking habits tend to form clusters within the network, indicating that smokers are more inclined to have connections with other smokers, particularly among family members and close friends, with family ties showing a more pronounced impact.

In the analysis of former smokers (table 5), female sex significantly reduces the likelihood of being a former smoker (Model 1, OR = 0.33; CI: 0.22, 0.48, *p* < 0.001; Model 3, OR = 0.32; CI: 0.22, 0.47, *p* < 0.001). Conversely, advancing age substantially increases the probability of being a former smoker (Model 1, OR = 1.76; CI: 1.41, 2.19, *p* < 0.001; Model 3, OR = 1.75; CI: 1.40, 2.19, *p* < 0.001). Among the ego characteristics, only age shows a significant effect (Model 1, OR = 0.65; CI: 0.44, 0.96, *p* < 0.05; Model 3, OR = 0.56; CI: 0.38, 0.82, *p* < 0.05). This suggests that unlike in the case of current smokers where both family and close friends’ smoking statuses were influential, for former smokers, only the presence of close friends who are former smokers significantly influences one’s own former smoker status (Model 3, OR = 2.84; CI: 1.08, 7.44, *p* < 0.05).

**Table 5.** Multilevel logistic regression models explaining alters’ former smoking status. This table presents multilevel regression models predicting whether alters are former smokers. The models consider individual attributes, network characteristics and both combined. The findings indicate that close friends who are former smokers significantly increase the likelihood of an alter being a former smoker, while family members play a lesser role in cessation. Additionally, assortativity of smokers and former smokers, network components and degree centralization show a significant impact, suggesting that more diverse and centralized networks may support smoking cessation.

	Model 0 (‘intercept’)		Model 1 (‘attributes’)		Model 2 (‘network’)		Model 3 (‘full’)	
	**OR (CI**)	***p***	**OR (CI**)	***p***	**OR (CI**)	***p***	**OR (CI**)	***p***
alter sex [ref. = male]			**0.33**	**<0.001**			**0.32**	**<0.001**
			**0.22, 0.48**				**0.22, 0.47**	
alter age (mean centred, scaled)			**1.76**	**<0.001**			**1.75**	**<0.001**
			**1.41, 2.19**				**1.40, 2.19**	
alter education [ref. = lower education]			0.89	0.565			0.92	0.694
			0.60, 1.32				0.62,1.38	
alter marital status [ref. = single]			1.49	0.111			1.43	0.161
			0.91, 2.44				0.87, 2.35	
ego sex [ref. = male]			1.05	0.899			1.04	0.910
			0.48, 2.28				0.50, 2.16	
ego age (mean centred, scaled)			**0.65**	**0.030**			**0.56**	**0.003**
			**0.44, 0.96**				**0.38 ,0.82**	
ego education [ref. = lower education]			0.88	0.732			0.94	0.857
			0.43, 1.80				0.47, 1.87	
ego marital status [ref. = single]			0.82	0.699			0.80	0.653
			0.31, 2.20				0.30, 2.13	
ego employment [ref. = unemployed]			1.82	0.132			1.76	0.138
			0.83, 3.98				0.83, 3.71	
acquaintance former smoker [ref. = other]			0.68	0.497			1.03	0.964
			0.22, 2.09				0.34, 3.12	
simple friend former smoker [ref. = other]			0.66	0.426			0.83	0.724
			0.23, 1.85				0.30, 2.33	
close friend former smoker [ref. = other]			2.27	0.097			**2.84**	**0.034**
			0.86, 5.95				**1.08, 7.44**	
family member former smoker [ref. = other]			1.55	0.314			1.93	0.118
			0.66, 3.62				0.85, 4.39	
ego alter meeting frequency [ref. = less than weekly]					1.31	0.156	1.12	0.566
					0.90, 1.92		0.75, 1.68	
alter degree (mean centred, scaled)					1.08	0.477	1.02	0.841
					0.88, 1.33		0.82, 1.28	
alter betweenness (mean centred, scaled)					1.02	0.839	1.07	0.526
					0.84, 1.23		0.87, 1.30	
network components					**1.17**	**0.044**	**1.24**	**0.005**
					**1.00, 1.36**		**1.07, 1.43**	
network degree centralization					10.37	0.098	**24.19**	**0.028**
					0.65, 166.24		**1.41, 415.64**	
network density					11.43	0.065	10.99	0.073
					0.86, 152.14		0.80, 150.83	
assortativity (smokers)					**2.67**	**0.041**	**4.00**	**0.006**
					**1.04, 6.85**		**1.50, 10.67**	
assortativity (former smokers)					**6.34**	**<0.001**	**4.59**	**0.005**
					**2.35, 17.09**		**1.60, 13.13**	
intercept	0.08	**<0.001**	0.07	**<0.001**	0.01	**<0.001**	0.00	**<0.001**
	0.05, 0.12		0.02, 0.26		0.00, 0.06		0.00, 0.05	
num.obs	1622		1622		1622		1622	
num.groups: ego_id	76		76		76		76	
ICC	0.32		0.29		0.30		0.24	
AIC	1074.405		1016.314		1066.666		1007.600	
BIC	1085.188		1097.185		1120.58		1131.602	
log likelihood	−535.203		−493.157		−523.333		−480.800	
deviance	1070.405		986.314		1046.666		961.600	
marginal *R*^2^/conditional *R*^2^	0.000/0.324		0.155/0.404		0.060/0.338		0.231/0.417	

The *assortativity score* for former smokers serves as a potent predictor of clustering behaviour, as evidenced in Model 2 (OR = 6.34; CI: 2.35, 17.09, *p* < 0.001) and Model 3 (OR = 4.59; CI: 1.60, 13.13, *p* < 0.05), indicating a tendency for former smokers to form clusters within social networks based on similar smoking statuses. Additionally, our analysis extends to the assortativity score for current smokers, which also demonstrates significance (Model 2, OR = 2.67; CI: 1.04, 6.85, *p* < 0.05; Model 3, OR = 4.00; CI: 1.50, 10.67, *p* < 0.05). This finding highlights the influence of current smokers within networks of former smokers, suggesting that in networks characterized by prevalent smoking, there could be an intensified peer influence or social pressure that potentially encourages former smokers to resume smoking.

The situation of former smokers, particularly when compared with the one of current smokers, underscores the significance of network metrics. In this vein, the finding regarding network degree centralization is noteworthy: it indicates that in networks characterized by a few individuals having significantly more connections than others, there exists a higher likelihood of alters being former smokers (Model 3, OR = 24.19; CI: 1.41, 415.64, *p* < 0.05). This outcome suggests the pivotal role of certain network members who may exert influence on the smoking behaviours of former smokers, potentially aiding in their cessation efforts or, conversely, contributing to a higher risk of relapse. The broad CI for this estimate, however, warrants a cautious interpretation of these findings. Furthermore, the analysis highlights the relevance of the number of components (Model 2, OR = 1.17; CI: 1.00, 1.36, *p* < 0.05; Model 3, OR = 1.24; CI: 1.07, 1.43, *p* < 0.05), indicating that engagement with diverse social groups can have an impact on smoking behaviour.

Table 6 showcases the findings pertaining to non-smokers. Sex plays a significant role, with being female markedly elevating the likelihood of being a non-smoker (Model 1, OR = 2.49; 95% CI: 1.97, 3.16, *p* < 0.001; Model 3, OR = 2.56; CI: 2.01, 3.25, *p* < 0.001). Alter age, alter and ego education or alter and ego marital status are not statistically significant. This means that in the case of non-smokers, the sociodemographic profile has a lower importance in contrast to network characteristics. Among non-smokers, the employment status of the ego is notably significant, indicating that alters associated with employed egos are less likely to smoke compared to those connected with unemployed egos (Model 1, OR = 0.63; 95% CI: 0.40, 0.99, *p* < 0.05; Model 3, OR = 0.63; CI: 0.40, 0.99, *p* < 0.05). Additionally, ego age is significant in Model 3, suggesting that older egos may exert a greater influence on the non-smoking behaviour of their alters (OR = 1.38; CI: 1.10, 1.73, *p* < 0.05). In other words, as ego age increases, the likelihood of influencing their social circle towards non-smoking behaviours appears to grow, underscoring the role of older individuals in promoting healthier behaviours within their networks.

**Table 6.** Multilevel logistic regression models explaining alters’ non-smoking status. This table provides multilevel logistic regression models for predicting whether alters are non-smokers. Key predictors include non-smoking family members and social network structure, such as assortativity of non-smokers and network components. The models show that non-smokers are more likely to be embedded in smoke-free social environments, reinforcing the clustering of similar behaviours within personal networks.

	Model 0 (‘intercept’)		Model 1 (‘attributes’)		Model 2 (‘network’)		Model 3 (‘full’)	
	**OR (CI**)	***p***	**OR (CI**)	***p***	**OR (CI**)	***p***	**OR (CI**)	***p***
alter sex [ref. = male]			**2.49**	**<0.001**			**2.56**	**<0.001**
			**1.97, 3.16**				**2.01, 3.25**	
alter age (mean centred, scaled)			1.14	0.052			1.13	0.069
			1.00, 1.30				0.99, 1.29	
alter education [ref. = lower education]			1.11	0.417			1.05	0.731
			0.86, 1.43				0.81, 1.35	
alter marital status [ref. = single]			0.91	0.533			0.93	0.613
			0.68, 1.22				0.69, 1.25	
ego sex [ref. = male]			0.98	0.919			1.09	0.693
			0.63, 1.52				0.71, 1.68	
ego age (mean centred, scaled)			1.25	0.053			**1.38**	**0.005**
			1.00, 1.57				**1.10, 1.73**	
ego education [ref. = lower education]			1.32	0.208			1.30	0.228
			0.86, 2.02				0.85, 1.99	
ego marital status [ref. = single]			1.02	0.954			1.06	0.853
			0.56, 1.83				0.58, 1.93	
ego employment [ref. = unemployed]			**0.63**	**0.043**			**0.63**	**0.046**
			**0.40, 0.99**				**0.40, 0.99**	
acquaintance non-smoker [ref. = other]			1.51	0.304			1.35	0.462
			0.69, 3.31				0.61, 3.00	
simple friend non-smoker [ref. = other]			1.08	0.793			1.06	0.847
			0.59, 1.98				0.58, 1.95	
close friend non-smoker [ref. = other]			1.28	0.433			1.06	0.840
			0.69, 2.34				0.58, 1.94	
family member non-smoker [ref. = other]			**1.83**	**0.011**			**1.64**	**0.035**
			**1.15, 2.93**				**1.04, 2.61**	
ego alter meeting frequency [ref. = less than weekly]					0.90	0.389	0.94	0.665
					0.71, 1.15		0.73, 1.22	
alter degree (mean centred, scaled)					0.92	0.256	0.90	0.167
					0.80, 1.06		0.77, 1.05	
alter betweenness (mean centred, scaled)					0.98	0.814	0.98	0.804
					0.86, 1.12		0.86, 1.13	
network components					0.95	0.289	**0.89**	**0.009**
					0.86, 1.04		**0.81, 0.97**	
network degree centralization					0.55	0.501	0.27	0.109
					0.10, 3.12		0.06, 1.33	
network density					**0.18**	**0.037**	0.23	0.053
					**0.04, 0.90**		0.05, 1.02	
assortativity (smokers)					1.29	0.579	1.10	0.834
					0.53, 3.12		0.44, 2.76	
assortativity (non-smokers)					**5.61**	**<0.001**	**4.91**	**<0.001**
					**2.46, 12.80**		**2.10, 11.47**	
intercept	1.69	**<0.001**	1.07	0.855	4.89	**0.015**	4.29	**0.022**
	1.36, 2.11		0.53, 2.14		1.36, 17.53		1.24, 14.89	
num.obs	1622		1622		1622		1622	
num.groups: ego_id	76		76		76		76	
ICC	0.18		0.13		0.17		0.11	
AIC	2056.081		1987.822		2029.770		1957.717	
BIC	2066.864		2068.693		2083.684		2081.72	
log likelihood	−1026.041		−978.911		−1004.885		−955.859	
deviance	2052.081		1957.822		2009.770		1911.717	
marginal *R*^2^/conditional *R*^2^	0.000/0.176		0.121/0.237		0.046/0.204		0.168/0.262	

The presence of a non-smoking family member significantly increases the odds of an individual being a non-smoker (Model 1, OR = 1.83; 95% CI: 1.15, 2.93, *p* < 0.05; Model 3, OR = 1.64; CI: 1.04, 2.61, *p* < 0.05), underscoring the role of immediate family in influencing smoking behaviours, akin to the patterns observed in smoker models. The *assortativity score* for non-smokers registers as highly significant (Model 2, OR = 5.61; CI: 2.46, 12.80, *p* < 0.001; Model 3, OR = 4.91; CI: 2.10, 11.47, *p* < 0.001), signifying a pronounced tendency for non-smokers to form clusters within their social networks. This pronounced assortativity among non-smokers accentuates the impact of social networks, indicating that non-smokers are likely influenced by the smoking habits of their peers. Furthermore, the significance of network components suggests that as the number of distinct network components increases, the likelihood of being a non-smoker slightly decreases (Model 3, OR = 0.89; CI: 0.81, 0.97, *p* < 0.05).

In addition to the multilevel logistic regression models, we executed standard logistic GLMs. The outcomes for all three categories of smoking behaviour (smokers, former smokers and non-smokers) are documented in electronic supplementary material, tables S36, S44 and S52. The comparison of these regression models reveals consistent results concerning the alters’ sex (e.g. for smokers: OR = 0.60; CI: 0.47, 0.77, *p* < 0.001), age (e.g. for smokers: OR = 0.70; CI: 0.61, 0.80, *p* < 0.001) and assortativity score (e.g. for smokers: OR = 0.21; CI: 0.07, 0.65, *p* < 0.05) across specific smoking statuses. To accommodate for the absence of random ego intercepts, these models also factored in the proportion of smokers, former smokers or non-smokers excluding the alter of interest, revealing a positive and significant effect across all three smoking categories.

## 4. Discussion

Our study analysed the impact of family members, friends and acquaintances with different smoking habits on the smoking habits of people located in a rural community using a PNA design. Deploying multilevel logistic regression models, we aimed to predict the smoking status of alters using various variables, such as alters’ sex, education, age, relationship status (being single or in a relationship), betweenness centrality, meeting frequency between ego and alter (at least weekly or less than weekly), together with the assortativity in smoking behaviour for level one. For level two, we used egos’ sex, education, age, employment status (employed or unemployed), type of ego (family members, close friends and acquaintances) and the smoking status of the ego. Network measurements included alter degree, alter betweenness, number of components, degree centralization, and density.

The primary finding of our research emphasizes the influence that social networks—consisting of friends and family members with diverse smoking patterns—have on the smoking behaviours of individuals. Specifically, the presence of smokers within one’s network markedly increases the likelihood of an individual being a smoker. Conversely, having family members or close friends who are non-smokers or former smokers significantly boosts the odds of an individual being a non-smoker or a former smoker, respectively. Our analysis indicates that both close friends and family members play significant roles, yet their impact can differ depending on the smoking status in question—be it current smoking status, former smoking status or the likelihood of being a non-smoker. The strong influence of family members suggests that smoking habits circulate more within the family instead of close friends’ group.

Our current results are related to findings from other research, highlighting that individuals with family members who smoke are more likely to adopt similar smoking habits [68]. While multiple studies reported that the impact of close friends is larger than that of the family [31,69], our results show similar trends but in the case of former smokers. For current smokers, our results highlight the higher importance of family. These contrasting results should be carefully considered as most studies related to the impact of friendship and family in smoking behaviour are (i) focused mainly on adolescents and (ii) not discriminating between profiles of smoking habits (smokers, former smokers or non-smokers).

For former smokers, the analysis reveals that having close friends who are former smokers significantly increases the likelihood of an individual also being a former smoker. The effect of family in this case is not statistically significant. In contrast, the influence of family members appears to be more pronounced in the models for non-smokers. While having a family member who is a non-smoker significantly increases the likelihood of an individual being a non-smoker, the effect size is smaller compared to the models for current smokers.

Secondary results relate to alters’ sociodemographic characteristics. Our analysis reveals sex and age as critical determinants of smoking behaviour, with females less likely to smoke or be former smokers and older individuals more inclined towards being former smokers. These results are in line with studies suggesting that the prevalence of smoking is higher in males than females [70]. Furthermore, our results are similar to the ones found in [71] and [72] where older adults who had ever smoked cigarettes had decided to quit.

Another important result relates to the effect of assortative mixing. Across all models, assortativity scores emerge as a very important predictor, highlighting the tendency of individuals to associate with peers sharing similar smoking behaviours. In other words, alters with a higher proportion of direct network neighbours that are smokers, former smokers or non-smokers, compared to the overall network proportion, have a higher chance of being classified as smokers, former smokers or non-smokers. This clustering effect within social networks—where smokers gravitate towards smokers, and non-smokers form connections with non-smokers—highlights the reinforcement of smoking habits through social connections.

In light of these results, we recommend that policies address both individual social characteristics and the social interactions shaping these behaviours. The study’s examination of various smoking patterns highlights the need for policies tailored to specific smoker categories. This is particularly important because the assortativity score for former smokers is significantly related to current smokers, suggesting that the presence of current smokers in a social network can hinder cessation efforts. Therefore, network interventions should be designed to strengthen connections between non-smokers and former smokers, or between non-smokers and current smokers, creating a supportive environment that encourages quitting or maintaining cessation.

Our study has several limitations. The first one is related to the design of PNA itself. Given that the data rely on the ego’s own perception of alters, the reliance on ego-reported data about alters’ attributes and relationships may be prone to potential inaccuracies such as the false consensus effect [73,74], or due to data recall [75] or social desirability biases [76,77]. In this sense, we limited the number of elicited alters to nearly 25, trying to help the ego identify them based on how frequently they communicate and how emotionally close they are. Future research should also consider cases of overlapping alters (i.e. individuals who appear in multiple personal networks) and instances where egos also serve as alters in other networks. It will be important to control for variations or discrepancies in reported information, such as when an ego identifies themselves as a former smoker, while others, for whom this ego is an alter, report them as a non-smoker.

Given the fact that our study is based on cross-sectional data, we cannot distinguish between social selection and social influence within the context of smoking behaviours. Specifically, we cannot determine whether being part of a network with higher rates of smoking leads individuals to adopt smoking behaviours (social influence), or if individuals who already have a preference for smoking are more likely to associate with others who share similar smoking habits (social selection). Collecting longitudinal data may differentiate between these effects.

Another potential limitation of this study stems from the participant selection process. The link-tracing method was initiated with six seeds, chosen to reflect diversity in sex, age, education, employment status, sector and personal income. Notably, smoking status was not a factor in selecting either the initial seeds or subsequent participants through referral chains. Moreover, the sample size of 76 personal networks may introduce some bias. Future research should explore the link-tracing data collection process, with particular attention to the recruitment of egos (e.g. examining differences in outcomes between investigator-driven and respondent-driven selection methods). Nevertheless, the consistency of our findings with prior studies supports our conclusions regarding the influence of family members [68], as well as the effects of age [71,72] and sex [70].

In terms of the qualitative aspects of relationships—specifically, whether two nodes are family members—our study design accounted only for the relationships between egos and alters. Family ties between alters, whether consanguineal or affinal, were not included in the models, as this information was unavailable (e.g. the proportion of a smoking alter’s direct network neighbours who are both family members and smokers). Such data could offer valuable insights into how assortativity operates within alters’ sub-networks, allowing for the creation of assortativity classes that could be compared (e.g. the assortativity effect of smoking family members versus that of smoking close friends). Additionally, collecting data on alters from the same ego at multiple points in time could enhance our understanding of the consistency in the composition of the nominated set of alters.

Despite these limitations, this study’s unique contribution is to examine the role of social networks in influencing smoking behaviours among adults in a rural setting using a PNA approach. This study differentiates itself by focusing not only on current smokers but also on former smokers and non-smokers, providing a comprehensive view of smoking behaviours within personal networks. This approach offers a novel perspective on addressing smoking in rural communities, where social ties, along with sociodemographic characteristics may have a particularly pronounced impact on health behaviours.

Given that adults who live in rural areas are mostly overlooked [26], further research should be expanded to other similar areas in an effort to increase the understanding of smoking behaviour in these areas. Also, it is important that future research investigate whether the findings of the current study may be applied to health-related behaviours that are not only tied to smoking.

The electronic supplementary material, including the code and other relevant details, as well as the data, are openly available [78], thereby enhancing the reproducibility of the study and facilitating further research.

## Data Availability

The dataset analysed in the current study and the R code (as electronic supplementary material) are openly available and deposited in the Zenodo General Repository [78].
